# Association between oxidative balance score and sarcopenia in US adults: NHANES 2011–2018

**DOI:** 10.3389/fnut.2024.1342113

**Published:** 2024-04-24

**Authors:** Weihang Xu, Dongmei Mu, Yuehui Wang, Ying Wang, Changcong Wang, Xinyue Zhang

**Affiliations:** ^1^Division of Clinical Research, The First Hospital of Jilin University, Changchun, China; ^2^School of Public Health, Jilin University, Changchun, China; ^3^Department of Geriatrics, The First Hospital of Jilin University, Changchun, China; ^4^Coll Comp Sci & Technol, Jilin University, Changchun, China

**Keywords:** oxidative stress, oxidative balance score, sarcopenia, NHANES, skeletal muscle

## Abstract

**Background:**

Sarcopenia, a condition characterized by diminished skeletal muscle mass, strength, and function, accompanied by inflammation and oxidative stress, remains an area of limited exploration concerning its correlation with the Oxidative Balance Score (OBS).

**Methods:**

Leveraging data from the 2011–2018 National Health and Nutrition Examination Survey (NHANES), we meticulously examined 16 dietary and four lifestyle factors to derive the OBS. Adjusting appendicular skeletal muscle mass (ASM) by body mass index (BMI) served as the designated marker for sarcopenia. To scrutinize the association between OBS and sarcopenia, we conducted weighted logistic regression and engaged in sensitivity analysis. Furthermore, we implemented subgroup analysis and interaction tests to gain comprehensive insights into the relationship across diverse populations.

**Results:**

In a sample comprising 6,677 individuals aged 20–59, logistic regression illuminated a negative association between OBS and sarcopenia [OR = 0.942 (0.920, 0.964), *p* < 0.001]. Robust associations were also discerned between diseases and both dietary and lifestyle OBS. Subgroup analysis unveiled a more pronounced negative association in older, married/living with partner or more educated individuals. Moreover, this association persisted in populations grappling with comorbidities such as hypertension, diabetes, cancer, and arthritis.

**Conclusion:**

Our study posits a perceptible link between OBS and the prevalence of sarcopenia among American adults.

## 1 Introduction

Sarcopenia is defined as a disease characterized by reduced skeletal muscle mass associated with decreased strength and function (1, 2). As an age-related disease, the disease occurs at all ages and results in adverse out-comes including recurrent falls, disability, fractures, frailty, poor quality of life, increased hospitalizations and mortality (3–7). Furthermore, studies have demonstrated that sarcopenia is associated with several diseases, including type 2 diabetes (8), osteoarthritis (9), cardiovascular disease (10–12), cirrhosis (13), some inflammatory disease (14). While the underlying causes of sarcopenia are not fully understood, most researchers believe that it results from a combination of factors, including changes in metabolic profile, malnutrition, prolonged sedentary time, hormonal, epigenetic and cellular changes (15–17). Oxidative stress and inflammation are crucial pathophysiological features of sarcopenia. Excessive oxidative stress impairs mitochondrial quality control, damaging mitochondrial membranes, DNA, proteins, and lipids. This damage compromises the structure and function of the mitochondria, which leads to increased production of reactive oxygen species (ROS) and further oxidative stress. This vicious cycle can result in muscle cell dysfunction and progressive loss of muscle mass (18–21).

Under normal physiological conditions, humans maintain a balance between oxidants and the antioxidant system. However, pro-oxidants can induce oxidative stress through the generation of reactive oxygen species (ROS) or the reduction of the defense activity of the antioxidant system (22). If the production of ROS exceeds the scavenging capacity of the antioxidant response system, it triggers oxidative damage, leading to cellular degeneration and reduced physiological function (23). Oxidative stress is implicated in several diseases (24), including metabolic syndrome (25), cardiovascular disease (CVD) (26), cancer (27, 28), Alzheimer’s disease (29). Due to the complex biological interactions between pro-oxidants and antioxidants, reliance on a single oxidative stress-related factor for assessment of overall oxidative homeostasis *in vivo* is inadequate. To overcome this limitation, researchers developed the Oxidative Balance Score (OBS), which calculated an individual’s pro-oxidant and total antioxidant exposure to assess overall antioxidant status, reflecting the impact of diet and lifestyle on the oxidative/antioxidant system (22, 30). Higher OBS scores indicate greater anti-oxidant exposure (31). Several studies have shown that OBS was negatively correlated with diabetes (32), cardiovascular disease (33), chronic kidney disease (34), osteoarthritis (34), non-alcoholic fatty liver disease (35), depression (36), and telomere length (31). OBS is a useful measure for assessing the cumulative impact of oxidative stress-related exposures in the study of chronic disease (37).

Since oxidative stress is closely associated with sarcopenia, it is crucial to use indicators that assess the overall oxidative/antioxidant system to evaluate the risk of the disease. Many current studies focus only on examining the relationship between inflammatory biomarkers such as CRP, IL-6, and TNF-α and muscle mass (14, 17, 38, 39). However, none of the studies have investigated the relationship of OBS with sarcopenia. Therefore, our study utilized data from the National Health and Nutrition Examination Survey (NHANES) with the aim of evaluating whether there is an association between the OBS index and sarcopenia in U.S. adults using a cross-sectional study approach. We hypothesize that an elevated OBS Index is associated with a lower risk of developing sarcopenia.

## 2 Materials and methods

### 2.1 Study population

This study was a cross-sectional study and the data were primarily obtained from the National Health and Nutrition Examination Survey (NHANES), a program that is widely used by scholar-researchers to assess the health and nutrition status of adults and children in the United States. The program consists of five main data sets collected through interviews and physical examinations, including demographic, dietary, medical assessment, laboratory, and questionnaire data (40). Each participant completed questionnaires and examinations at the Mobile Examination Center (MEC) over a one-year period to obtain important study variables, including physical condition, physiological and biochemical indices, and dietary habits at specific times of the year. Data collection is approved by the National Center for Health Statistics Research Ethics Review Board, and written informed consent was obtained from participants (41).

**Figure 1:**
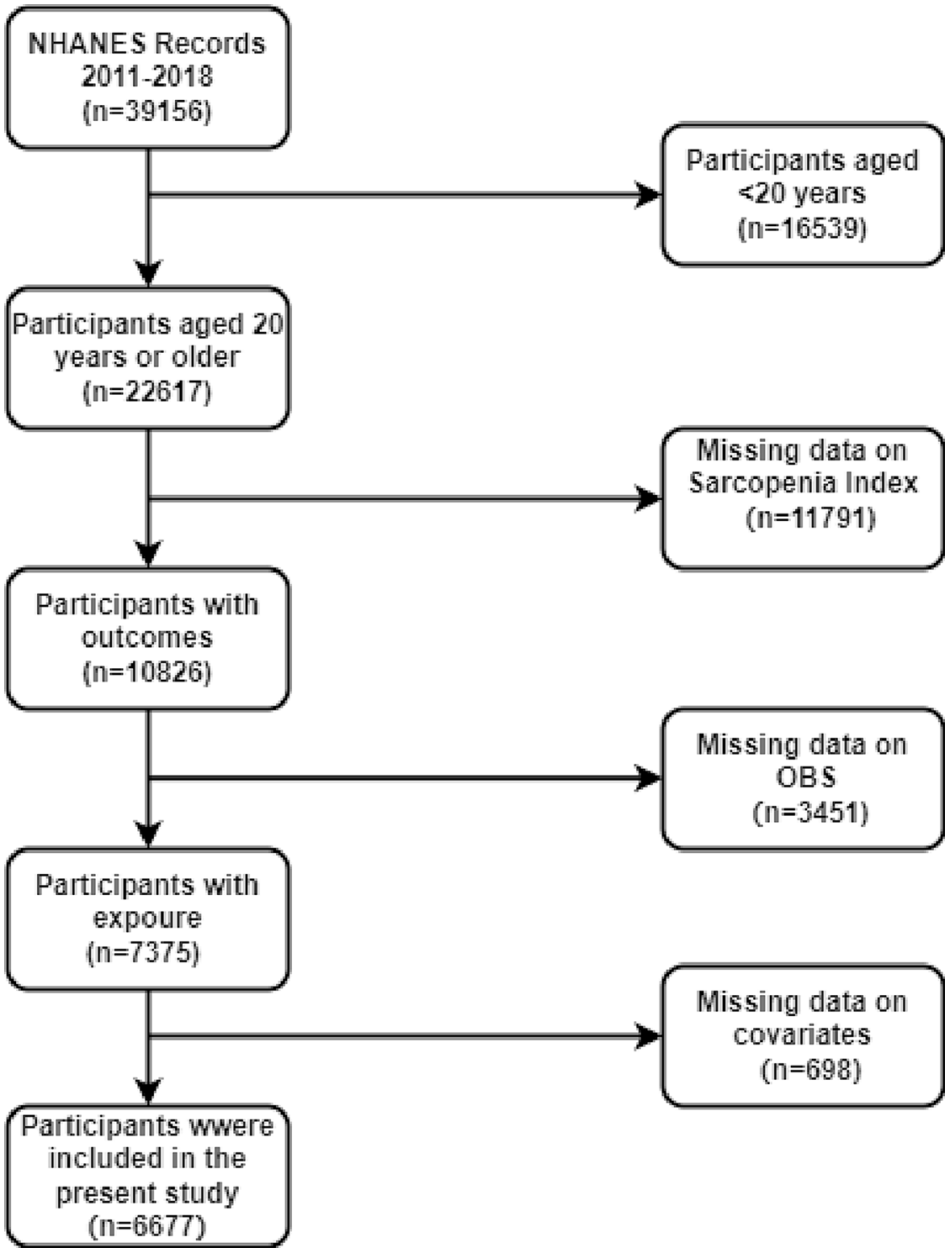
Flowchart of the sample selection from NHANES 2011–2018.

In this study, data from four cycles of the NHANES surveys (2011–2018) were used, which included a total of 39,156 participants. Individuals younger than 20 years (*n* = 16,539) were excluded, as shown in Figure 1. Furthermore, participants with missing sarcopenia index data (*n* = 11,791), incomplete OBS data (*n* = 3,451), and missing covariates (*n* = 698) were omitted. As a consequence, only 6,677 participants were eligible for complete case analysis.

### 2.2 Outcome variable

In this study, sarcopenia was the primary outcome variable, defined as a condition related to muscle mass. To measure sarcopenia, we assessed total appendicular lean mass (ALM) via Dual-energy X-ray absorptiometry (DEXA) within the NAHNES program. Those ineligible for DEXA testing due to a height exceeding 192.5 cm, weight exceeding 136.4 kg, or pregnancy were excluded.

Sarcopenia was objectively defined by the use of the sarcopenia index, which was a composite of appendicular skeletal muscle mass and BMI. The index was calculated by dividing the total mass of the appendicular skeletal muscle (in kg) by the BMI (in kg/m^2^) (42, 43). For the diagnosis of sarcopenia, the index (also known as sarcopenia index, BMI-adjusted ALM or ALMBMI) had different cut-off values for men (0.789) and women (0.512), according to guidelines from the National Institutes of Health (FNIH). These cut-off values were used to diagnose sarcopenia (43).

### 2.3 Exposure variable

The study required the calculation of the Oxidative Balance Score (OBS) through a compilation of pro-oxidant and antioxidant elements derived from dietary and lifestyle factors (31). The dietary-based OBS components comprising 16 nutrients, including dietary fiber, carotene, riboflavin, niacin, vitamin B6, total folate, vitamin B12, vitamin C, vitamin E, calcium, magnesium, zinc, copper, selenium, total fat and iron were obtained from 24-h dietary recall interviews. The interviews consisted of two sessions: the first one in person and the second one by phone 3–10 days later. However, this study only analyzed the data from the first interview as the main predictor. This was to ensure the reliability of the self-reported dietary intake and avoid confounding effects of variations in other physiological and biochemical markers and health conditions measured during the interview owing to the long time gap.

The lifestyle-based OBS components evaluated lifestyle factors such as physical activity, BMI, alcohol consumption and smoking status. Among them, total fat, iron, BMI, alcohol consumption, and smoking status were considered pro-oxidant, while the remaining variables were considered antioxidant.

To determine the physical activity score, we adhered to the NHANES guidelines and calculated metabolic equivalent (MET) scores. The scores included work-related (vigorous and moderate) and leisure-time physical activities (vigorous and moderate), as well as walking or bicycling for transportation. MET scores = weekly frequency of each physical activity * duration of each physical activity* each physical activity suggested MET Scores. The smoking status was evaluated using serum cotinine, a major metabolite of nicotine (44).

Following the method of calculating OBS by Zhang et al. (31), alcohol consumption was divided into three groups, heavy drinkers (≥15 g/d for women and ≥ 30 g/d for men), non-heavy drinkers (0–15 g/d for women and 0–30 g/d for men), and non-drinkers, who were assigned 0, 1, and 2 points, respectively.

Then, the components were grouped by gender and then divided into three groups on a weighted basis according to their tertiles, with antioxidants scoring 0–2 in groups 1–3, and pro-oxidants scoring 2–0 in groups 1–3, respectively (31, 45). The sum of OBSs varied from 0 to 40 points. For this study, we used total OBS, dietary OBS, and lifestyle OBS as variables. The detailed plan of OBS can be found in Table 1.

**Table 1:** Oxidative balance score calculation.

OBS components	Property	Male			Female		
		0	1	2	0	1	2
Dietary OBS components							
Dietary fiber (g/ day)	A	<13.40	13.4–21.9	≥21.9	<10.6	10.6–18.1	≥18.1
Carotene (mcg/day)	A	<452	452–1,481	≥1,481	<430.	430–1786	≥1786
Riboflavin (mg/day)	A	<1.857	1.857–2.854	≥2.854	<1.41	1.41–2.18	≥2.18
Niacin (mg/day)	A	<24.04	24.04–36.16	≥36.16	<16.56	16.56–24.76	≥24.76
Vitamin B6 (mg/day)	A	<1.76	1.76–2.8	≥2.80	<1.26	1.26–2.01	≥2.01
Total folate (mcg/day)	A	<329	329–532	≥532	<245	245–401	≥401
Vitamin B12 (mcg/day)	A	<3.39	3.39–6.46	≥6.46	<2.28	2.28–4.36	≥4.36
Vitamin C (mg/day)	A	<28.50	28.5–89.9	≥89.90	<27.80	27.8–80.7	≥80.70
Vitamin E (mg/day)	A	<6.83	6.83–11.49	≥11.49	<5.51	5.51–9.49	≥9.49
Calcium (mg/day)	A	<802	802–1,284	≥1,284	<643	643–1,011	≥1,011
Magnesium (mg/day)	A	<273	273–400	≥400	<217	217–317	≥317
Zinc (mg/day)	A	<9.88	9.88–15.49	≥15.49	<7.00	7.00–10.77	≥10.77
Copper (mg/day)	A	<1.04	1.04–1.56	≥1.56	<0.85	0.85–1.28	≥1.28
Selenium (mcg/day)	A	<107.20	107.2–160.5	≥160.50	<74.30	74.30–111.40	≥111.40
Total fat (g/day)	P	≥115.2	74.6–115.2	<74.60	≥84.86	55.88–84.86	<55.88
Iron (mg/day)	P	≥12.39	12.39–18.91	<18.91	≥13.97	9.05–13.97	<9.05
Lifestyle OBS components							
Physical activity (MET-minute/week)	A	<3,360	3,360–6,720	≥6,720	<1,680	1,680–4,760	≥4,760
Alcohol (g/day)	P	≥30	0–30	None	≥15	0–15	None
Body mass index (kg/m^2^)	P	≥30	25.60–30	<25.6	≥30.1	24.3–30.1	<24.3
Cotinine (ng/mL)	P	≥1.26	0.017–1.26	<0.017	≥0.07	0.01–0.07	<0.01

A stood for the antioxidant, P for the pro-oxidant, MET for the metabolic equivalent.

### 2.4 Covariates

Covariates in this study included demographic, examination, laboratory, and questionnaire data. In terms of demographics, we included age (<30, 30–40, 40–50, >50), gender, race (Mexican American, Hispanic, non-Hispanic white, non-Hispanic black, or others), education level (less than high school, high school or general educational development, more than high school), marital status (never married, married/living with partner, separated/divorced/widowed), poverty–income ratio (PIR; <1.3, 1.3–3.5, 3.5). For medical history, we considered diabetes, cardiovascular disease (CVD; including coronary heart disease, angina, heart failure, heart attack, and stroke), arthritis, cancer, and chronic kidney disease (CKD). In addition, we incorporated high-density lipoprotein cholesterol (HDL-C; mg/dL), total cholesterol (TC; mg/dL) and total energy intake (kcal). More details on variable collection methods can be found in the NHANES Survey Methods and Analysis Guide.

### 2.5 Statistical analysis

We took into account the recommended weights in our statistical analysis due to the specificities of NHANES, which gathered data based on a complex multistage cluster survey design, and used one-fourth of the examination weight as the new sample weight in line with NHANES’ weight selection guidelines. We present a statistical characterization of the baseline variable based on the presence or absence of Sarcopenia. Continuous variables were expressed as mean ± standard deviation, while categorical variables were presented as number (percentage). ANOVA tests were used for comparing continuous variables, and chi-square tests were employed to examine statistical differences in categorical variables between groups.

Furthermore, we conducted weighted multivariate logistic regression analyses to evaluate the association between OBS and sarcopenia by adjusting for potential confounders and calculating odds ratios (ORs) and 95% confidence intervals (CIs). Meanwhile, to investigate any linear correlation trends between OBS and sarcopenia, we converted OBS from a continuous variable to a categorical variable (quartiles). Survey weights were considered in all regression analyses. In addition, as OBS can be subdivided into dietary OBS and lifestyle OBS, and may also be strongly linked to sarcopenia, we conducted distinct studies investigating the association between the two types of OBS and sarcopenia. In the extended models, Model 1 was a crude model without covariate-adjusted; Model 2 was adjusted for age, gender, race/ethnicity, education level, PIR; Model 3 was Model 2 plus HDL-C, total cholesterol, total energy intake, diabetes, CVD, CKD, hypertension, arthritis, and cancer.

Subgroup analyses on the relationship between OBS and sarcopenia were executed in accordance with model 3, with stratified factors including gender (male/female), age (<30, 30–40, 40–50, >50), race (Mexican American, other Hispanic, non-Hispanic white, non-Hispanic black, and other), PIR (<1.3, 1.3–3.5, >3.5), educational level (<High school, High school, >High school), marital status (never married, married/living with partner, separated/divorced/widowed), hypertension (yes/no), diabetes (yes/no), cardiovascular disease (yes/no), and chronic kidney disease (yes/no), cancer (yes/no), arthritis (yes/no). Interaction tests were used to examine the consistency of the relationships between the different subgroups. Identical analyses were conducted for dietary OBS and lifestyle OBS and the results were then presented through forest plots.

Additionally, the stability of the findings was examined through sensitivity analyses. The association between OBS and sarcopenia was re-evaluated using multivariate logistic regressions after excluding extreme values of BMI below 15 kg/m^2^ and above 50 kg/m^2^. Secondly, we utilized ALMBMI, which refers to BMI-adjusted ALM, to evaluate muscle mass. We assessed the correlation between OBS and muscle mass through multiple linear regressions. Furthermore, we performed sensitivity analyses under unweighted conditions to assess the stability of the association between OBS and sarcopenia.

Statistical significance was determined when *p* < 0.05. All statistical analyses were conducted using R software (version 4.1.1).

## 3 Results

### 3.1 Characteristics of the study population

The characteristics of individuals grouped by sarcopenia are presented in Table 2. The study included 6,677 subjects, with a mean age (standard deviation) of 39.0 (11.71) years, comprising 3,401 (50.94%) males and 3,276 (49.06%) females. A total of 473 participants were diagnosed with Sarcopenia. Individuals with sarcopenia demonstrated a higher probability of being older, Mexican American or non-Hispanic white, having higher BMI, lower educational levels, lower income, reduced physical activity, lower HDL-C levels, and lower total energy intake (all *p* < 0.05), as compared to those without sarcopenia. OBS was lower in those with sarcopenia (17.456 ± 6.644) than in those without (20.606 ± 7.087), as were dietary OBS and lifestyle OBS. Furthermore, patients with sarcopenia were more prone to accompanying comorbidities such as diabetes, cardiovascular disease, arthritis, cancer, hypertension and chronic kidney disease.

**Table 2:** Characteristics of study participants by muscle status (*n* = 6,677).

Characteristic	Level	Total (*n* = 6,677)	Sarcopenia (*n* = 473)	Without Sarcopenia (*n* = 6,204)	*p*-value
Age(year), Mean ± SD		38.992 ± 11.710	42.236 ± 12.129	38.796 ± 11.656	< 0.001
Sex, n (%)	Male	3,401 (50.94)	237 (50.11)	3,164 (51.00)	0.225
	Female	3,276 (49.06)	236 (49.89)	3,040 (49.00)	
Race, n (%)	Mexican American	894 (13.39)	160 (33.83)	734 (11.83)	< 0.001
	Other Hispanic	623 (9.33)	70 (14.80)	553 (8.91)	
	Non-Hispanic White	2,570 (38.49)	131 (27.70)	2,439 (39.31)	
	Non-Hispanic Black	1,348 (20.19)	30 (6.34)	1,318 (21.24)	
	Other Race	1,242 (18.60)	82 (17.34)	1,160 (18.70)	
PIR, n (%)	<1.3	2016 (30.19)	188 (39.75)	1828 (29.46)	< 0.001
	1.3–3.5	2,364 (35.41)	171 (36.15)	2,193 (35.35)	
	>3.5	2,297 (34.40)	114 (24.10)	2,183 (35.19)	
Education, n (%)	<High school	943 (14.12)	121 (25.58)	822 (13.25)	< 0.001
	High school	3,656 (54.76)	265 (56.03)	3,391 (54.66)	
	>High school	2078 (31.12)	87 (18.39)	1991 (32.09)	
Marital status, n (%)	Never married	3,949 (59.14)	102 (21.56)	1714 (27.63)	0.027
	Married/living with partner	912 (13.66)	284 (60.04)	3,665 (59.07)	
	Separated/Divorced/Widowed	1,165 (17.45)	87 (18.39)	825 (13.30)	
Drinking status, n (%)	Heavy drinkers	1,165 (17.45)	61 (12.90)	1,104 (17.79)	0.010
	Non-heavy drinkers	623 (9.33)	22 (4.65)	601 (9.69)	
	Non-drinkers	4,889 (73.22)	390 (82.45)	4,499 (72.52)	
Diabetes mellitus, n (%)	No	6,221 (93.17)	393 (83.09)	5,828 (93.94)	< 0.001
	Yes	456 (6.83)	80 (16.91)	376 (6.06)	
CVD, n (%)	No	6,451 (96.62)	440 (93.02)	6,011 (96.89)	< 0.001
	Yes	226 (3.38)	33 (6.98)	193 (3.11)	
Arthritis, n (%)	No	5,828 (87.28)	386 (81.61)	5,442 (87.72)	0.007
	Yes	849 (12.72)	87 (18.39)	762 (12.28)	
Cancer, n (%)	No	6,434 (96.36)	443 (93.66)	5,991 (96.57)	0.029
	Yes	243 (3.64)	30 (6.34)	213 (3.43)	
Hypertension, n (%)	No	4,863 (72.83)	283 (59.83)	4,580 (73.82)	< 0.001
	Yes	1814 (27.17)	190 (40.17)	1,624 (26.18)	
CKD, n (%)	No	6,120 (91.66)	415 (87.74)	5,705 (91.96)	0.006
	Yes	557 (8.34)	58 (12.26)	499 (8.04)	
BMI (kg/m^2^), Mean ± SD		28.380 ± 6.391	35.329 ± 8.076	27.960 ± 6.024	<0.001
TEM (minute/week), Mean ± SD		4964.943 ± 4157.445	4397.502 ± 3848.772	4999.198 ± 4173.114	0.013
Cotinine (ng/mL), Mean ± SD		57.212 ± 125.826	45.280 ± 122.442	57.932 ± 125.999	0.071
HDL-C (mg/dL), Mean ± SD		53.484 ± 16.066	48.178 ± 13.381	53.804 ± 16.159	< 0.001
TC (mg/dL), Mean ± SD		191.278 ± 40.542	196.401 ± 43.984	190.969 ± 40.307	0.079
Total energy intake (kcal), Mean ± SD		2277.059 ± 1006.810	2066.153 ± 864.425	2289.790 ± 1013.412	< 0.001
Total OBS, Mean ± SD		20.426 ± 7.100	17.456 ± 6.644	20.606 ± 7.087	< 0.001
Diet OBS, Mean ± SD		15.986 ± 6.720	13.771 ± 6.328	16.119 ± 6.720	< 0.001
Life OBS, Mean ± SD		4.441 ± 1.671	3.685 ± 1.567	4.486 ± 1.666	< 0.001

PIR, income to poverty ratio; CVD, Cardiovascular disease; CKD, Chronic Kidney Disease; BMI, body mass index; MET, metabolic equivalent; HDL-C, high-density lipoprotein cholesterol; TC, total cholesterol; OBS, oxidative balance score.

Furthermore, after dividing the OBS into quartiles, it was observed that individuals in the top OBS quartile were more likely to be female, Mexican American, and non-Hispanic white in comparison to those in the bottom OBS quartile. Additionally, participants in the top OBS quartile showed higher levels of education and income in terms of socioeconomic status. Participants in the top OBS quartile were less likely to develop several diseases, including diabetes, cardiovascular disease, arthritis, hypertension, chronic kidney disease, and sarcopenia, indicating a healthier state (Supplementary Table S1).

### 3.2 Association between OBS and sarcopenia

As demonstrated in Table 3, we examined the relationship between OBS and risk of sarcopenia based on weighted logistic regression. When OBS was treated as a continuous variable, we observed that it was significantly associated with a lower incidence of developing sarcopenia in both crude model [OR = 0.938 (0.923, 0.954), *p* < 0.001] and fully adjusted model [OR = 0.942 (0.920, 0.964), *p* < 0.001]. When considering OBS as a categorical variable, the fully adjusted model (model 3) indicated that the highest quartile of OBS was linked to lower risk of sarcopenia prevalence when compared to the lowest quartile of OBS [OR = 0.389 (0.253, 0.599), *p* < 0.001]. This association persisted through various models. However, the second quartile of OBS did not demonstrate a significant association with sarcopenia risk in fully adjusted model. A trend test revealed a significant association between OBS and the risk of developing sarcopenia (*p* for trend<0.001).

**Table 3:** Relationship between total OBS and Sarcopenia.

Total OBS	Model 1		Model 2		Model 3	
	OR (95%CI)	*p*-value	OR (95%CI)	*p*-value	OR (95%CI)	*p*-value
Continuous^a^	0.938 (0.923, 0.954)	<0.001	0.936 (0.918, 0.954)	<0.001	0.942 (0.920, 0.964)	<0.001
Quartiles^b^						
Q1	Ref		Ref		Ref	
Q2	0.670 (0.508, 0.885)	0.007	0.675 (0.499, 0.912)	0.014	0.745 (0.531, 1.044)	0.096
Q3	0.444 (0.298, 0.660)	< 0.001	0.419 (0.274, 0.640)	< 0.001	0.486 (0.303, 0.778)	0.005
Q4	0.322 (0.230, 0.452)	< 0.001	0.329 (0.229, 0.470)	< 0.001	0.389 (0.253, 0.599)	< 0.001
*p* for trend		<0.001		<0.001		<0.001

a Total OBS was treated as a continuous variable.

b Total OBS was treated as a four-categorical variable.

Model 1 was a crude model without covariate-adjusted; Model 2 was adjusted for age, gender, race/ethnicity, education level, PIR, marital status; Model 3 was Model 2 plus HDL-C, TC, total energy intake, diabetes, CVD, CKD, hypertension, arthritis, and cancer. Abbreviation: PIR, income to poverty ratio; CVD, Cardiovascular disease; CKD, Chronic Kidney Disease; HDL-C, high-density lipoprotein cholesterol; TC, total cholesterol; OBS, oxidative balance score.

### 3.3 Association between dietary OBS/lifestyle OBS and sarcopenia

After categorizing OBS into dietary and lifestyle OBS, we assessed their association with sarcopenia (Table 4). The results indicate that dietary OBS was statistically significant after adjusting for all confounders [OR = 0.961 (0.937, 0.986), *p* = 0.003], and a negative association with the disease was observed. Compared to lowest quartile of dietary OBS, the highest quartile of dietary OBS was significantly and negatively associated with sarcopenia [OR = 0.452 (0.280, 0.732), *p* = 0.003].

**Table 4:** Relationship between dietary/lifestyle OBS and Sarcopenia.

	Model 1		Model 2		Model 3	
	OR (95%CI)	*p*-value	OR (95%CI)	*p*-value	OR (95%CI)	*p*-value
Dietary OBS						
Continuous^a^	0.949 (0.932, 0.966)	<0.001	0.948 (0.93, 0.967)	<0.001	0.961 (0.937, 0.986)	0.005
Quartiles^b^						
Q1	Ref		Ref		Ref	
Q2	0.878 (0.64, 1.206)	0.425	0.902 (0.643, 1.266)	0.554	1.007 (0.723,1.403)	0.966
Q3	0.642 (0.46, 0.895)	0.011	0.627 (0.442, 0.890)	0.012	0.770 (0.514, 1.155)	0.210
Q4	0.352 (0.239, 0.519)	<0.001	0.360 (0.239, 0.542)	<0.001	0.452 (0.280, 0.732)	0.003
*p* for trend		<0.001		<0.001		0.004
Lifestyle OBS						
Continuous^c^	0.750 (0.706, 0.797)	<0.001	0.738 (0.687, 0.794)	<0.001	0.745 (0.688, 0.807)	<0.001
Quartiles^d^						
Q1	Ref		Ref		Ref	
Q2	0.597 (0.437, 0.817)	0.002	0.565 (0.397, 0.804)	0.003	0.555 (0.388, 0.794)	0.003
Q3	0.447 (0.341, 0.585)	<0.001	0.412 (0.307, 0.551)	<0.001	0.436 (0.320, 0.595)	<0.001
Q4	0.109 (0.060, 0.195)	<0.001	0.114 (0.062, 0.212)	<0.001	0.122 (0.064, 0.234)	<0.001
*p* for trend		<0.001		<0.001		<0.001

a Dietary OBS was treated as a continuous variable.

b Dietary OBS was treated as a four-categorical variable.

c Lifestyle OBS was treated as a continuous variable.

d Lifestyle OBS was treated as a four-categorical variable.

Model 1 was a crude model without covariate-adjusted; Model 2 was adjusted for age, gender, race/ethnicity, education level, PIR, marital status; Model 3 was Model 2 plus HDL-C, TC, total energy intake, diabetes, CVD, CKD, hypertension, arthritis, and cancer. Abbreviation: PIR, income to poverty ratio; CVD, Cardiovascular disease; CKD, Chronic Kidney Disease; HDL-C, high-density lipoprotein cholesterol; TC, total cholesterol; OBS, oxidative balance score.

In the final adjusted model, lifestyle OBS was significantly associated with a reduced risk of developing sarcopenia [OR = 0.745 (0.688, 0.807), *p* < 0.001]. In addition, with the second, third and fourth quartiles all showing significant statistical associations, greater adherence to lifestyle OBS was associated with a lower risk of developing sarcopenia. Trend tests demonstrated that both OBS were linked to a statistically significant decreasing trend in the risk of disease prevalence (*p* = 0.004 and *p* < 0.001, respectively).

### 3.4 Subgroup analysis

To investigate the link between OBS and sarcopenia in diverse populations, we conducted analyses stratified by subgroups including sex, age, race, PIR, educational level, marital status, hypertension, diabetes, cardiovascular disease, chronic kidney disease, cancer and arthritis (Table 5). Our findings suggested that the degree of correlation varies among different populations. We found a significant interaction between education level and sarcopenia (*p* for interaction = 0.008), and the protective effect of association between OBS and sarcopenia was greater for those with higher education [OR = 0.887 (0.838, 0.940), *p* < 0.001] than for those with a high school education or less [OR = 0.946 (0.925, 0.968), *p* < 0.001]. There was also a significant interaction between arthritis and sarcopenia (*p* for interaction =0.027). The negative association effect of OBS with sarcopenia was significantly higher in individuals with arthritis [OR = 0.902 (0.850, 0.958), *p* = 0.002] compared to those without arthritis [OR = 0.951 (0.927, 0.976), *p* < 0.001]. For people over 50 years of age or married/living with partner, this indicator shows a significant protective effect. Individuals with comorbidities such as hypertension, diabetes, cancer were more protected from this association than those without such conditions though without significant interaction. Our results indicated that the association between OBS and sarcopenia remained negative in most subgroups, although no significant association was found in some subgroups.

**Table 5:** The association between total OBS and sarcopenia according to different variables.

Subgroup	No of participants	OR (95%CI)	*p*-value	*p* for interaction
Sex				0.971
Male	3,401	0.952 (0.926, 0.980)	0.002	
Female	3,276	0.921 (0.892, 0.951)	<0.001	
Age				0.279
<30	2043	0.945 (0.893, 0.999)	0.053	
30–40	1,670	0.981 (0.930, 1.040)	0.499	
40–50	1,617	0.961 (0.923, 1.000)	0.059	
>50	1,347	0.904 (0.859, 0.952)	<0.001	
Race				0.045
Mexican American	894	0.982 (0.944, 1.020)	0.355	
Other Hispanic	623	0.968 (0.915, 1.020)	0.260	
Non-Hispanic White	2,570	0.915 (0.876, 0.955)	<0.001	
Non-Hispanic Black	1,348	0.853 (0.766, 0.949)	0.006	
Other Race	1,242	0.972 (0.914, 1.030)	0.479	
PIR				0.407
<1.3	2016	0.953 (0.916, 0.991)	0.020	
1.3–3.5	2,364	0.948 (0.913, 0.984)	0.008	
>3.5	2,297	0.918 (0.871, 0.967)	0.003	
Educational Level				0.008
<High school	943	0.999 (0.939, 1.064)	0.979	
High school	3,656	0.946 (0.925, 0.968)	< 0.001	
>High school	2078	0.887 (0.838, 0.940)	< 0.001	
Marital status				0.613
Never married	1726	0.957 (0.907, 1.010)	0.117	
Married/living with partner	3,949	0.931 (0.898, 0.964)	< 0.001	
Separated/Divorced/Widowed	912	0.950 (0.890, 1.014)	0.128	
Hypertension				0.408
No	4,863	0.952 (0.925, 0.979)	0.002	
Yes	1814	0.922 (0.879, 0.967)	0.002	
Diabetes				0.883
No	6,221	0.951 (0.926, 0.976)	<0.001	
Yes	456	0.902 (0.854, 0.953)	<0.001	
CVD				0.682
No	6,451	0.944 (0.922, 0.967)	<0.001	
Yes	226	0.915 (0.811, 1.032)	0.164	
CKD				0.554
No	6,120	0.937 (0.914, 0.961)	<0.001	
Yes	557	0.992 (0.944, 1.043)	0.755	
Cancer				0.906
No	6,434	0.943 (0.920, 0.968)	<0.001	
Yes	243	0.879 (0.79, 0.978)	0.027	
Arthritis				0.027
No	5,828	0.951 (0.927, 0.976)	<0.001	
Yes	849	0.902 (0.850, 0.958)	0.002	

After being adjusted for age, gender, race/ethnicity, education level, PIR, marital status, HDL-C, total cholesterol, total energy intake, diabetes, CVD, CKD, hypertension, arthritis, and cancer. Abbreviation: PIR, income to poverty ratio; CVD, Cardiovascular disease; CKD, Chronic Kidney Disease; HDL-C, high-density lipoprotein cholesterol; TC, total cholesterol; OBS, oxidative balance score.

**Figure 2:**
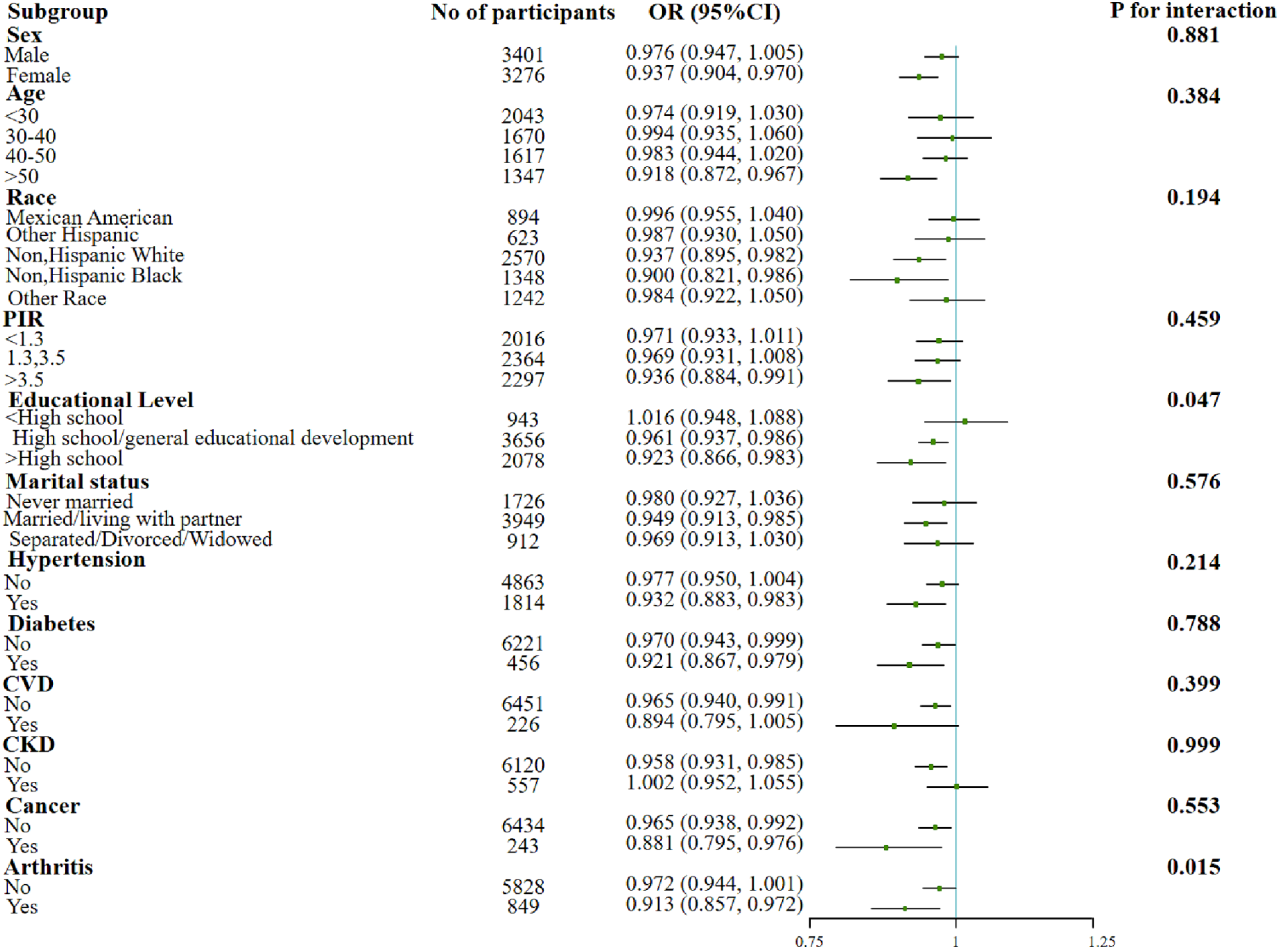
Forest plot for the effect of dietary OBS on sarcopenia according to different variables. After being adjusted for age, gender, race/ethnicity, education level, PIR, marital status, HDL-C, total cholesterol, total energy intake, diabetes, CVD, CKD, hypertension, arthritis, and cancer. Abbreviation: PIR, income to poverty ratio; CVD, Cardiovascular disease; CKD, Chronic Kidney Disease; HDL-C, high-density lipoprotein cholesterol; TC, total cholesterol; OBS, oxidative balance score.

**Figure 3:**
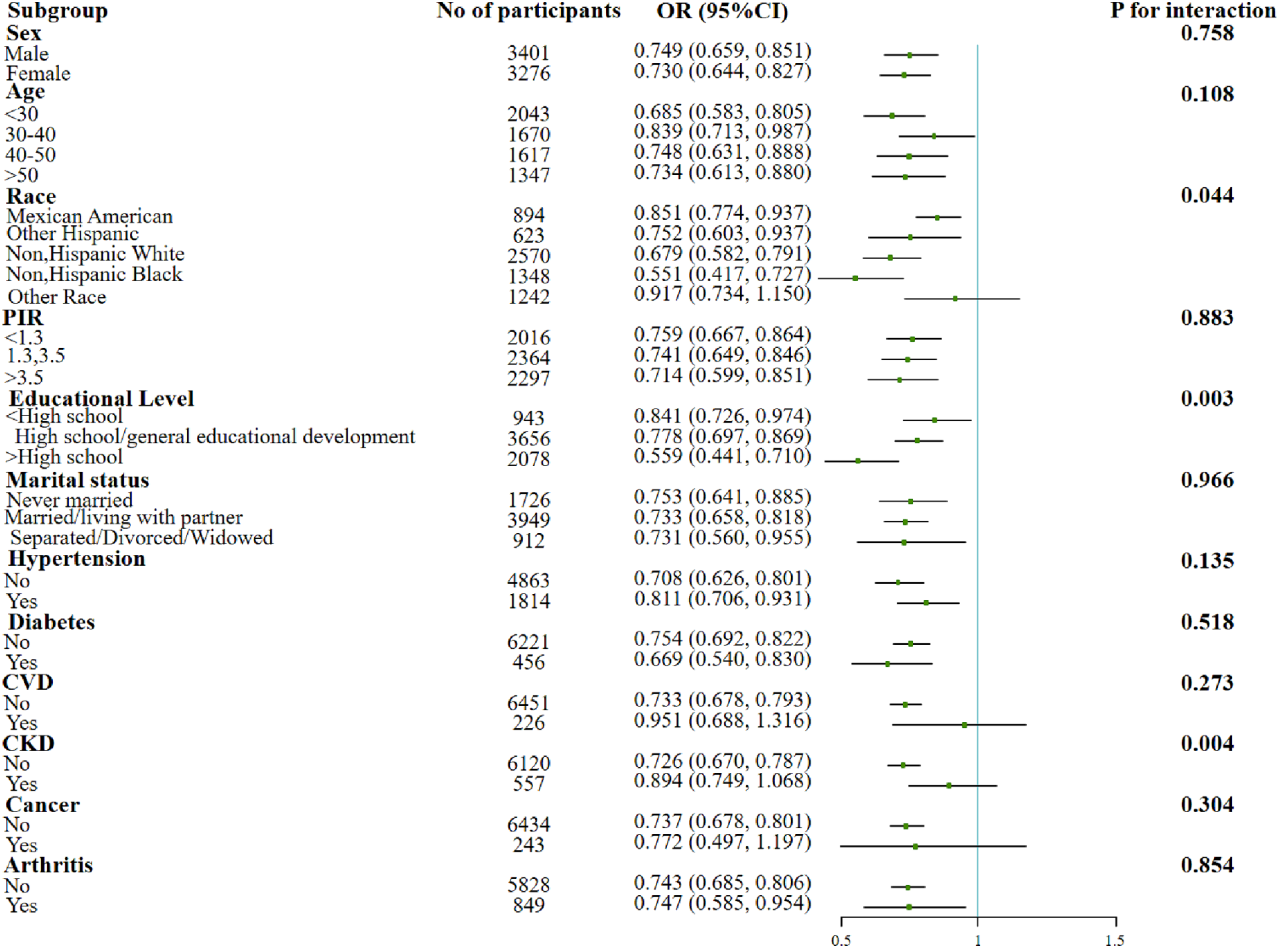
Forest plot for the effect of lifestyle OBS on sarcopenia according to different variables. After being adjusted for age, gender, race/ethnicity, education level, PIR, marital status, HDL-C, total cholesterol, total energy intake, diabetes, CVD, CKD, hypertension, arthritis, and cancer. Abbreviation: PIR, income to poverty ratio; CVD, Cardiovascular disease; CKD, Chronic Kidney Disease; HDL-C, high-density lipoprotein cholesterol; TC, total cholesterol; OBS, oxidative balance score.

In addition, significant interactions were also found in the education subgroups when examining the association between the dietary OBS or lifestyle OBS and sarcopenia. The protective effect of the associations was found to be better in more educated populations. Moreover, both indicators were negatively associated with sarcopenia in people with elder age, lower income, married/living with partner and co-morbid conditions such as hypertension, diabetes, and arthritis (Figures 2, 3).

### 3.5 Sensitivity analysis

We performed sensitivity analyses using multiple methods to determine the robustness of the results. Removal of BMI outliers (<15 kg/m^2^, *n* = 0) and (>60 kg/m^2^, *n* = 56; Supplementary Table S2) or under weighted condition did not significantly affect the results of correlation (Supplementary Table S3). In addition, when ALMBMI was used as a continuous variable to measure muscle mass, a significant positive association between OBS and muscle mass was observed after adjustment for confounders (*p* < 0.001; Supplementary Table S4). Higher OBS related to better muscle mass, indicating a lower risk of sarcopenia. The correlation be-tween OBS and muscle mass status would therefore be more effectively validated.

## 4 Discussion

To elucidate the association between OBS and sarcopenia, we conducted cross-sectional analyses on 6,677 individuals from the NHANES database. Our study revealed that total OBS, dietary OBS, and lifestyle OBS are all negatively correlated with the prevalence of sarcopenia. Furthermore, the high OBS category significantly reduced the risk of sarcopenia compared to the lowest category, even after accounting for potential confounding factors. Additionally, the link between OBS and sarcopenia was particularly pronounced in older age categories, lower incomes, higher education, and individuals suffering from hypertension, diabetes, cancer, and arthritis. In summary, a higher OBS score corresponded to a lower likelihood of developing sarcopenia. Our research also underscored the importance of maintaining a healthy diet and life-style, with notable implications for public healthcare.

Sarcopenia, an age-related muscle wasting disease, has become a serious public health concern. Its complexity defies easy explanation, despite many studies. Several lines of evidence implicate that oxidative stress and inflammation are linked to sarcopenia onset and development. Aging impairs muscle nutrition and mitochondrial function, boosting ROS levels. With aging, both muscle nutrition and mitochondrial function declined, boosting the levels of reactive oxygen species (ROS). The accumulation of ROS increases protein hydrolysis, resulting in loss of muscle mass (46–48). Elevated levels of inflammatory biomarkers, such as C-reactive protein (CRP), inter-leukin-6 (IL-6), and α-tumor necrosis factor (TNFα), have been implicated in promoting muscle loss and are associated with decreased muscle mass and strength in older adults. The detrimental impact of oxidative stress on muscle tissue is exacerbated by the increased expression of inflammatory cytokines (39, 49). Inflammatory diets may contribute to muscle loss by fostering oxidative stress, systemic inflammation, and reduced insulin (50). Consequently, maintaining low levels of systemic inflammation and reducing oxidative stress is pivotal in averting the risk of developing sarcopenia.

In this study, we explored the relationship between OBS and the risk of sarcopenia. OBS, functioning as a comprehensive measure of oxidative or antioxidant balance, has the potential to offer deeper insights into the overall oxidative stress status of the body. While OBS is constrained to external exposures like dietary and non-dietary lifestyle factors, multiple studies have demonstrated a correlation between OBS and inflammation markers (such as CRP) (51) and indicators of oxidative stress (such as F2-isoprostane) (52) and gamma glutamyl transferase, GGT (53), affirming the reliability of OBS in assessing oxidative homeostasis (35). Moreover, the emphasis on external exposures aligns with the daily lives of the general population, making it easier to provide relevant guidance to healthcare providers for enhancing population health. Our results indicated that higher oxidative balance score (OBS) might confer antioxidant advantage over pro-oxidant exposure and was significantly negatively associated with sarcopenia risk.

Our findings align with previous research indicating a correlation between dietary factors and sarcopenia. Diets abundant in fruits and vegetables are shown to prevent metabolic acidosis, diminish protein hydrolysis and amino acid catabolism, and lower the likelihood of sarcopenia (11). Diets high in fiber play a crucial role in reducing the risk of lower lean muscle mass (54). The Mediterranean diet is characterized by low levels of inflammation and antioxidants found in plant-based foods, healthy fats and whole grains, which have beneficial effects on muscle mass, physical function and the prevention of sarcopenia (55). On the flip side, the consumption of foods rich in saturated fat can be detrimental to muscle health. The risk of muscle loss may also be heightened by adhering to a Western dietary pattern, characterized by the frequent intake of flour, bread, potatoes, red meat, processed meat, eggs, and cheese (56).

Moreover, certain antioxidant and anti-inflammatory nutrients, such as vitamins E and C, omega-3 fatty acids, MUFA, PUFA, and caffeine, may contribute to a reduction in the prevalence of sarcopenia (57). The UK Biobank population-wide cross-sectional analysis study (58) and the Maastricht Sarcopenia Study (59) found a negative association between magnesium intake and sarcopenia, and older adults with sarcopenia had lower daily magnesium intakes. Another case–control study compared 66 older adults without decreased muscle mass with previously recruited adults with sarcopenia and found that selenium intake was significantly lower in the case group (60). In a cross-sectional analysis of 396,283 individuals from the UK Biobank, a higher in-take of calcium or potassium was associated with lower odds of having sarcopenia (58). Antioxidant-rich diets may alter the course of sarcopenia by modulating the phase angle (PA). As a measure of cell membrane integrity and cellular health, PA was positively correlated with dietary intake of antioxidants. Decreased PA values may indicate impaired cell membrane function, which is associated with muscle tissue degeneration and loss of muscle mass. Research supports the use of PA as a biomarker to predict sarcopenia. Lower PA values were associated with a higher risk of sarcopenia, which emphasizes the important role of antioxidant-rich diets in reducing the risk of this disease (61, 62).

Several studies also have examined the relationship between life-style and sarcopenia and muscle mass, and these variables were included in our OBS components. Two studies conducted in Korea have established a correlation between alcohol intake and a higher prevalence of sarcopenia among women (63, 64). Similar findings were reported in animal studies, indicating an association between alcohol consumption and skeletal muscle mass loss (65, 66). Alcohol caused a reduction in mammalian/rapamycin (mTOR) kinase activity inhibited overall protein synthesis, and led to reduced protein synthesis in skeletal muscle and dysregulated responses to anabolic stimuli in enzymatic or several anabolic stimuli (67–69). In addition, a study from the SarcoPhAge cohort found that individuals who smoked more had more severe sarcopenia (70), and Rom et al. (71) demonstrated that smoking contributes to the breakdown of muscle proteins by oxidative stress. In a study in mice, Nogueira et al. (72) illustrated that smoking-induced capillary deconditioning contributes to the decline in skeletal muscle function. Collectively, smoking and chronic alcohol abuse interfered with muscle protein metabolism pathways, reducing skeletal muscle mass and in-creasing the risk of sarcopenia. Furthermore, these habits contributed to conditions such as periodontal disease, chronic lung, and liver disease, amplifying the risk of musculoskeletal disorders (73).

Furthermore, a wealth of studies indicated that physical activity, encompassing aerobic, resistance, and multimodal exercise, has the potential to enhance or sustain muscle function in older adults with sarcopenia (74). Numerous meta-analyses further underscored a positive correlation between engaging in any form of physical activity and a reduced likelihood of developing sarcopenia, promoting the preservation of muscle mass (70). The act of muscle cells producing reactive oxygen species (ROS) in response to exercise can serve to either trigger or inhibit various genes. This process enhances antioxidant defenses and suppresses the activation of inflammatory pathways, thereby fortifying the organism’s resistance to free radicals (75–77). Consequently, the most effective strategy for improving sarcopenia and averting disability involves regular physical activity coupled with appropriate nutritional support (78).

As age advances, there is a concurrent rise in visceral adipose tissue and a decline in muscle mass, culminating in the emergence of sarcopenia coexisting with obesity, commonly referred to as sarcopenic obesity. Indications of this condition encompass a heightened body mass index and diminished muscle mass (79, 80). Independently, obesity is linked with oxidative stress, a factor that intensifies with age and increasing body mass index (BMI), consequently progressively weakening the body’s antioxidant status (81). Both low-grade systemic inflammation and the accumulation of intramuscular fat contribute to mitochondrial dysfunction and impaired mitochondrial beta-oxidation. This cascade results in elevated lipid peroxidation, leading to the increased accumulation of lipid intermediates and reactive oxygen metabolites (ROM), thereby escalating inflammation, oxidative stress, and insulin resistance (IR) (82). Additionally, myocytes in this state release myokines, including the muscle growth-inhibiting hormone, irisin, TNF-alpha, and interleukin (IL), exacerbating IR in adipose tissue and other tissues (82, 83). A recent meta-analysis has revealed a correlation between sarcopenic obesity, especially in men, and a higher rate of all-cause mortality (84). Increased oxidative stress in sarcopenia was associated with high risk of cardiovascular disease in sarcopenic obesity (85). To preempt disease, it is imperative to adopt a healthy diet and engage in regular exercise. However, weight loss must be coupled with adequate protein intake and exercise, recognizing that obesity is a multifactorial condition, and weight loss alone can exacerbate muscle loss (86–88).

Our findings suggest that the protective effect of the association between OBS and sarcopenia is more pronounced in higher age brackets. Emphasizing the significance of this association in preventing and predicting disease within the geriatric cohort is crucial due to the heightened likelihood of gradual skeletal muscle dysfunction and atrophy in middle-aged and older individuals as a consequence of aging (2, 79). Both male and female OBS were significantly associated with sarcopenia, but the protective effect is stronger for females than for males. This difference may be due to the greater expression of antioxidant genes and enzyme activities in women, which may result in better maintenance of antioxidant capacity and a reduction in disease risk (89, 90). Moreover, a correlation between OBS and sarcopenia that is influenced by the level of education is also indicated in our findings. Educational attainment serves as an indicator of the ability to translate nutritional knowledge into improved dietary habits (91). It is plausible that individuals with higher education levels place greater emphasis on their diet and lifestyle to ensure good health. Similarly, married or partnered individuals tend to adopt healthier lifestyles and diets with their partner’s or family’s support, which links lower oxidative stress and disease risk (92).

Additionally, significant correlations between OBS and sarcopenia were observed in individuals with diabetes, hypertension, or arthritis. Our investigation revealed an increased prevalence of sarcopenia in individuals with these conditions, demonstrating a robust association with pro-inflammatory elements, oxidative stress, and other pathological mechanisms (9, 79, 93–96). Moreover, each of these diseases has been negatively associated with OBS in previous studies (34, 97–99). Therefore, assessing the antioxidant status of patients with diabetes, hypertension, or arthritis via OBS holds significant public health implications for preventing muscle osteoporosis.

Our study had several strengths. Firstly, to our knowledge, it represents the inaugural investigation assessing the relationship between OBS and sarcopenia within the general population. Secondly, we leveraged the NHANES database, renowned for its extensive sample size and accurate national representation. This enabled us to adjust for additional confounding variables, ensuring the reliability of our outcomes. Thirdly, our study differentiated itself by concentrating on OBS, a composite indicator rather than a single component, in order to comprehensively understand the complex relationships among factors and thoroughly examine the outcomes of interest.

However, our study presents several limitations. Firstly, being a cross-sectional analysis, it poses challenges in establishing a clear causal relationship. Additionally, the reliance on self-reported medical conditions introduces the potential for recall bias. To ascertain the precise relationship between OBS and the risk of sarcopenia, further prospective studies are essential. Secondly, the overall OBS score may either underestimate or overestimate pro-oxidant and antioxidant biological effects due to the equal weighting of all components. Additionally, there is a likelihood of threshold effects with high antioxidant doses, potentially inducing toxic pro-oxidant activity. Thirdly, despite our efforts to adjust for several confounders, the possibility of additional con-founding in our analyses and subgroup analyses cannot be entirely excluded. Due to the limitations of the NHANES database for obtaining data regarding the assessment of sarcopenia, such as age, height, and weight restrictions, this study focused only on the eligible adult population. However, for the high-risk elderly population, the use of OBS to assess disease risk is particularly important and the relevance of OBS to the disease in this age group necessitates further analysis.

## 5 Conclusion

Our study revealed a noteworthy association between OBS and the prevalence of sarcopenia in US adults. It underscored the importance of adopting an antioxidant-rich diet and implementing lifestyle modifications to mitigate the prevalence of sarcopenia. Nevertheless, it was crucial to acknowledge that factors such as education level, income situation, or the presence of chronic diseases could influence the observed results. While the practicality of using OBS to assess antioxidant levels in elderly individuals and those with diabetes, hypertension, or arthritis is potentially possible, further studies are warranted to delve deeper into these associations.

## Data availability statement

Publicly available datasets were analyzed in this study. This data can be found at: https://www.cdc.gov/nchs/nhanes/.

## Ethics statement

The studies involving humans were approved by the National Center for Health Statistics Research Ethics Review Board. The studies were conducted in accordance with the local legislation and institutional requirements. The participants provided their written informed consent to participate in this study. Written informed consent was obtained from the individual(s) for the publication of any potentially identifiable images or data included in this article.

## Author contributions

WX: Conceptualization, Data curation, Investigation, Software, Validation, Writing – original draft. DM: Conceptualization, Formal analysis, Funding acquisition, Investigation, Writing – original draft. YuW: Formal analysis, Funding acquisition, Investigation, Supervision, Writing – review & editing. YiW: Data curation, Funding acquisition, Investigation, Software, Supervision, Writing – review & editing. CW: Data curation, Methodology, Software, Writing – review & editing. XZ: Data curation, Supervision, Validation, Writing – review & editing.
